# Fast and Selective Post-polymerization Modification of Conjugated Polymers Using Dimethyldioxirane

**DOI:** 10.3389/fchem.2019.00123

**Published:** 2019-03-11

**Authors:** Emmanuel Reichsöllner, Adam Creamer, Shengyu Cong, Abby Casey, Simon Eder, Martin Heeney, Florian Glöcklhofer

**Affiliations:** ^1^Institute of Applied Synthetic Chemistry, TU Wien, Vienna, Austria; ^2^Department of Chemistry and Centre for Plastic Electronics, Imperial College London, London, United Kingdom

**Keywords:** organic electronics, conjugated polymers, post-polymerization modification, oxidation, sulfoxide, sulfone, PCDTBT, F8BT

## Abstract

Modification of functional groups attached to conjugated polymer backbones can drastically alter the material properties. Oxidation of electron-donating thioalkyl substituents to electron-withdrawing sulfoxides or sulfones is a particularly effective modification. However, so far, this reaction has not been studied for the modification of conjugated polymers used in organic electronics. Crucial questions regarding selectivity and reaction time waited to be addressed. Here, we show that the reaction is highly selective and complete within just a few minutes when using dimethyldioxirane (DMDO) for the oxidation of thioalkyl substituents attached to the well-investigated conjugated polymers poly(9-(1-octylnonyl)carbazole-*alt*-4,7-dithienylbenzothiadiazole) (PCDTBT) and poly(9,9-dioctylfluorene-*alt*-benzothiadiazole) (F8BT). The selectivity was confirmed by comparison with polymers obtained from pre-oxidized monomers and by control experiments using related polymers without thioalkyl substituents. Using DMDO, the oxidation yields acetone as the only side-product, which reduces the work-up to mere evaporation of solvents and excessive reagent. Our results show that this oxidation is an exciting method for the preparation of electron-deficient conjugated polymers. It may even allow the preparation of electron acceptors for solar cells directly from the electron donors.

## Introduction

Conjugated polymers are most frequently used in organic electronics, but the specific applications strongly depend on the properties of the polymer (Swager, 2017; Ibanez et al., 2018). Functional groups can be used to tune these properties (Guo et al., 2013); the desired functional groups can be introduced either by using appropriate monomers for polymerization or by post-polymerization modification. The latter can be achieved either by introducing new functional groups onto the polymer backbone (Crossley et al., 2017) or by modifying or substituting functional groups already present (Schelkle et al., 2014; Creamer et al., 2018). As the main advantage of post-polymerization modification, polymers with varying properties can be obtained from the same starting polymer by using different reagents for modification or by varying the degree or distribution of modification (statistical or, for example, surface modification). Furthermore, functional groups that would either impair polymerization or degrade during polymerization may be introduced. The modifications can be designed to slightly tune or optimize the optoelectronic properties (e.g., for tuning LUMO/LUMO offsets in solar cells) or to change them drastically (e.g., for turning electron donors into electron acceptors).

One such modification to drastically change the properties is the oxidation of electron donating thioalkyl substituents to electron withdrawing sulfones (or sulfoxides). Sulfone substituents have been reported to greatly lower the HOMO/LUMO energy levels of conjugated polymers, turning them into electron acceptors and improving their air stability (Zhang et al., 2009; Hubijar et al., 2013). To perform this modification on polymers, we considered dimethyldioxirane (DMDO) to be the most suitable reagent, as it yields acetone as the only side-product (González-Núñez et al., 2002), which can be easily removed by evaporation (in contrast to side-products of metal oxidants or larger organic oxidants). The easy removal of the side-products is highly beneficial for applications sensitive to impurities, including applications in organic electronics, and may even allow for post-processing modification, for example surface modification of polymer thin-films or nanoparticles. Using other reagents, such as *meta*-chloroperoxybenzoic acid (mCPBA) or Rozen's reagent, is reported to require washing with aqueous base and water for purification following the oxidation (Wei et al., 2014), which not only adds additional steps to the preparation but also potentially complicates solid state reactions.

We have previously shown that DMDO can be used for post-polymerization modification, but the polymers investigated (obtained by Cu(I)-catalyzed azide-alkyne cycloaddition polymerization) were of low molecular weight (M_n_ = 3.5–3.6 kDa, M_w_ = 7.2–7.5 kDa) and did not exhibit properties of conjugated polymers (Glöcklhofer et al., 2014, 2015); neither absorption at longer wavelengths nor electrical conductivity were observed. Therefore, it remained unclear whether DMDO can also be used for the modification of truly conjugated polymers. Furthermore, the selectivity of the reaction as well as the required reaction time have not been addressed so far, although—if fast and selective–such a modification of truly conjugated polymers would be particularly interesting for organic electronics, as it may turn polymers from preferentially hole-transporting to electron-transporting or from electron-donating to electron-accepting.

**Figure 1:**
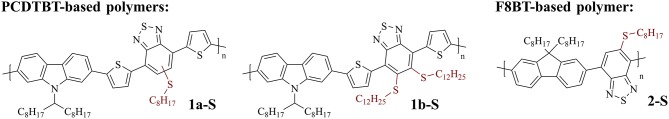
Conjugated polymers used to investigate the post-polymerization modification with DMDO. Left: PCDTBT-based polymers with one **(1a-S)** and two **(1b-S)** thioalkyl substituents attached to the benzothiadiazole units. Right: F8BT-based polymer with one thioalkyl substituent attached to the benzothiadiazole units **(2-S)**.

For the present work, two very well-investigated conjugated polymers were selected for modification to address these questions, PCDTBT and F8BT. Both polymers are frequently used in organic electronics, in particular for organic solar cell and transistor applications (Kim et al., 2004; Zaumseil et al., 2006; Beaupré and Leclerc, 2013). Thioalkyl substituents that enable the modification were attached to the benzothiadiazole units of the polymers (Figure 1). Polymers with one (**1a-S** and **2-S**) and two (**1b-S**) thioalkyl substituents at the benzothiadiazole units were selected.

It was our plan to (i) find suitable conditions for modification of the polymers (solvent, temperature, amount of DMDO), (ii) investigate the selectivity of the reaction (considering that thiophene, carbazole, and benzothiadiazole units are present, which are potentially prone to oxidation), and (iii) determine the required reaction time.

## Results

### PCDTBT-Based Polymers

**Figure 2:**
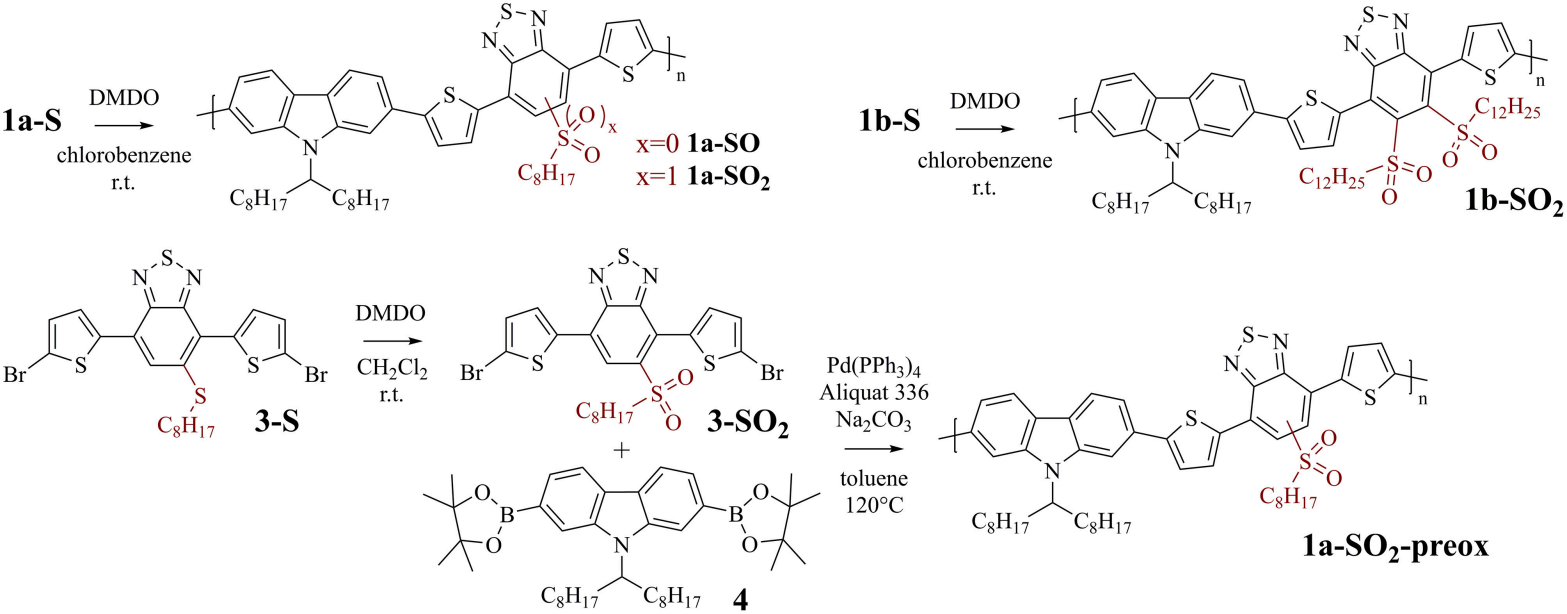
Top: Post-polymerization modification of PCDTBT-based polymers **1a-S** and **1b-S** to sulfoxide-substituted polymer **1a-SO** and sulfone-substituted polymers **1a-SO**_**2**_ and **1b-SO**_**2**_. Bottom: Synthesis of sulfone-substituted monomer **3-SO**_**2**_ and subsequent polymerization with **4**, affording sulfone-substituted polymer **1a-SO**_**2**_**-preox** for comparison with **1a-SO**_**2**_.

PCDTBT-based polymer **1a-S** (Figure 2, top left) was used for initial investigations focusing on reaction conditions and selectivity, as the carbazole, thiophene, and benzothiadiazole units of the polymer are all potentially prone to oxidation by DMDO (**Table 2** gives an overview of the tested reaction conditions).

**Figure 3:**
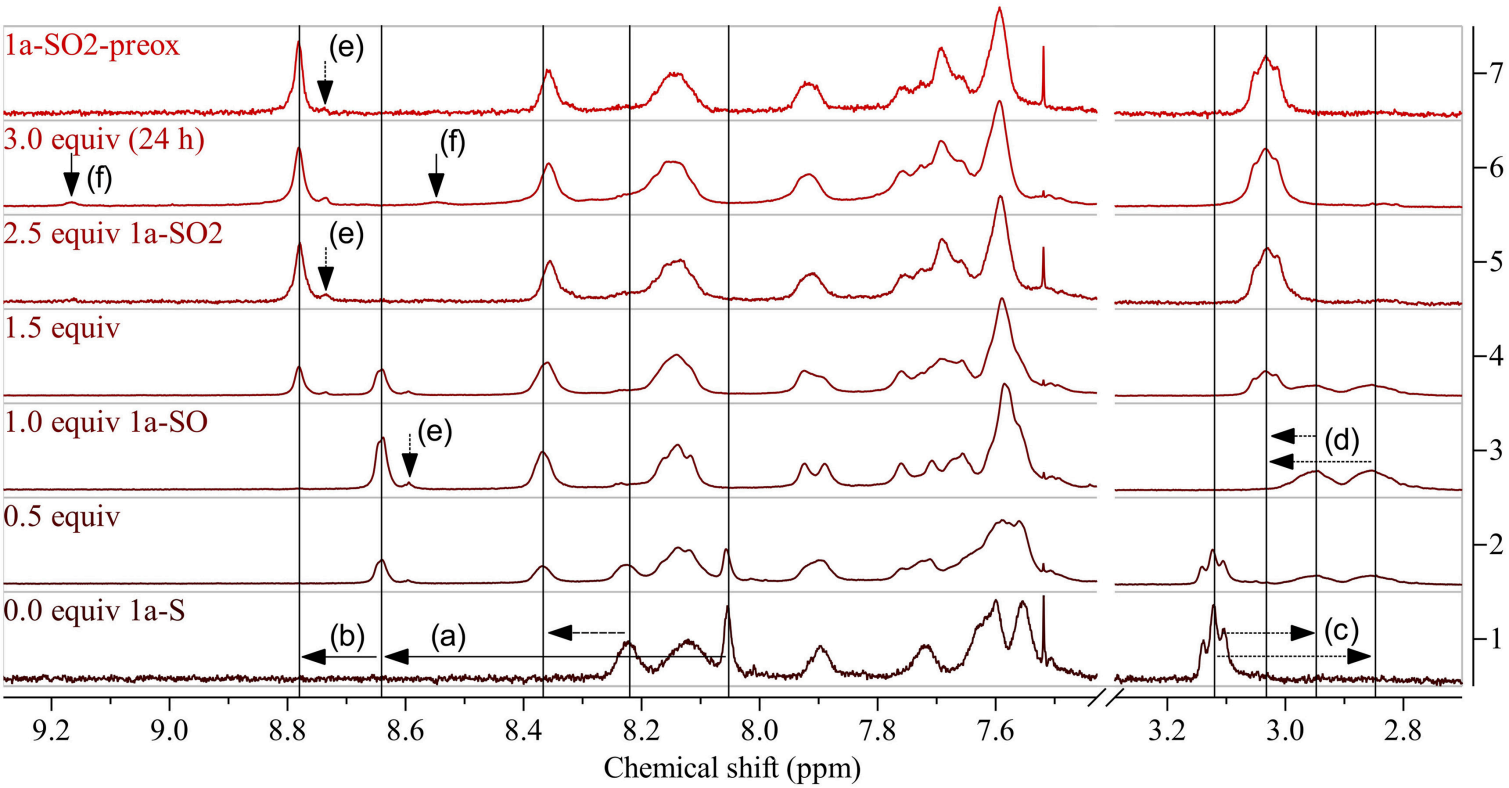
^1^H NMR spectra of **1a-S** (bottom) and corresponding polymers obtained by modification using increasing amounts of DMDO (bottom to top: 0.5 equiv, 1.0 equiv: **1a-SO**, 1.5 equiv, 2.5 equiv: **1a-SO**_**2**_). The selectivity of the modification was evaluated by treating the starting polymer with a large excess of DMDO for prolonged time [3.0 equiv (24 h)] and by comparison of **1a-SO**_**2**_ with **1a-SO**_**2**_**-preox** (top). Horizontal arrows show indicative shifts upon modification: (a) downfield shift of the peak of the benzothiadiazole proton upon oxidation to the sulfoxide and (b) further shift upon oxidation to the sulfone, (c) upfield shift and splitting of the peak of the aliphatic protons next to the thioalkyl sulfur upon oxidation to the sulfoxide and (d) downfield shift and reunification upon further oxidation to the sulfone. Vertical arrows indicate (e) peaks attributed to end groups and (f) side reactions. See Supplementary Material for full spectra.

The experiments showed that CH_2_Cl_2_ (which we used previously for modifications with DMDO) is not a suitable solvent for the modification of **1a-S**. The polymer precipitated during reaction, which resulted in side reactions indicated by the same ^1^H NMR peaks that we found later when treating the polymer with a large excess of DMDO for 24 h (Figure 3). Chlorobenzene was found to be a better solvent; testing different amounts of DMDO for the oxidation in this solvent revealed that 2.5 equivalents (equiv) per repeat unit of the polymer resulted in complete oxidation to **1a-SO**_**2**_. The oxidation resulted in significant shifts of the ^1^H NMR peaks of the protons next to the thioalkyl sulfur atom (Figure 3): the peak of the benzothiadiazole proton shifted from 8.05 to 8.78 ppm, the peak of the two aliphatic protons shifted from 3.12 to 3.03 ppm. The upfield shift of the aliphatic signal is counterintuitive but may be caused by steric effects. A similar upfield shift from 3.07–3.01 to 2.97–2.86 ppm was observed when oxidizing monomer **3-S** to **3-SO**_**2**_ (Figure 2). Comparison of the ^13^C NMR signal of the aliphatic carbon next to the thioalkyl sulfur, which should be less affected by steric effects, revealed that–upon oxidation–this peak is shifted downfield as expected, from 34.28 to 54.35 ppm. This corresponds well with the shift from 33.1 to 54.0 ppm observed by us in a similar oxidation (Glöcklhofer et al., 2014).

Interestingly, selective oxidation to the sulfoxide-substituted polymer **1a-SO** occurred when using 1.0 equiv of DMDO. A slightly smaller shift of the benzothiadiazole peak to 8.64 ppm was observed (Figure 3(a)); neither the benzothiadiazole peaks of **1a-S** nor **1a-SO**_**2**_ were present. Oxidation to the sulfoxide was confirmed by the splitting of the aliphatic peak into two peaks at 2.95 and 2.85 ppm (Figure 3(c)), a result of the two aliphatic protons becoming diastereotopic. Oxidation with 0.5/1.5 equiv resulted in the presence of the benzothiadiazole peaks of both **1a-SO** and **1a-S** / **1a-SO**_**2**_ and **1a-SO** in the expected ratio of 1:1. Treating the polymer with 3.0 equiv for 24 h resulted in the appearance of two small additional peaks at 9.17 and 8.55 ppm (Figure 3(f), solid vertical arrows), as mentioned above. This is attributed to slow side reactions occurring at other sites of the polymer that are prone to oxidation.

To confirm the selectivity of the modification of **1a-S** to **1a-SO**_**2**_ under the optimized conditions, we prepared polymer **1a-SO**_**2**_**-preox** for comparison using monomers **3-SO**_**2**_ and **4** (Figure 2, bottom). The ^1^H NMR spectra of **1a-SO**_**2**_ and **1a-SO**_**2**_**-preox** turned out to be identical, except for a small peak next to the benzothiadiazole peak, which was slightly larger for **1a-SO**_**2**_ (Figure 3(e), dotted vertical arrows). We attributed this peak to end groups of the polymers, as the corresponding peak was also present in the spectrum of **1a-SO** and as the size of the peak did not increase when treating the polymer with 3.0 equiv DMDO for 24 h. As this meant **1a-SO**_**2**_**-preox** must have a significantly higher average molecular weight than the other polymers, we performed GPC measurements, confirming our assumption (Table 1).

**Table 1:** GPC results of PCDTBT-based polymers **1a-S**, **1a-SO**, **1a-SO**_**2**_, and **1a-SO**_**2**_**-preox**.

**Polymer**	**M_**n**_ [kDa]**	**M_**w**_ [kDa]**	**Ð**
**1a-S**	23	52	2.3
**1a-SO**	23	50	2.2
**1a-SO**_**2**_	22	49	2.2
**1a-SO**_**2**_**-preox**	53	97	1.8

**Figure 4:**
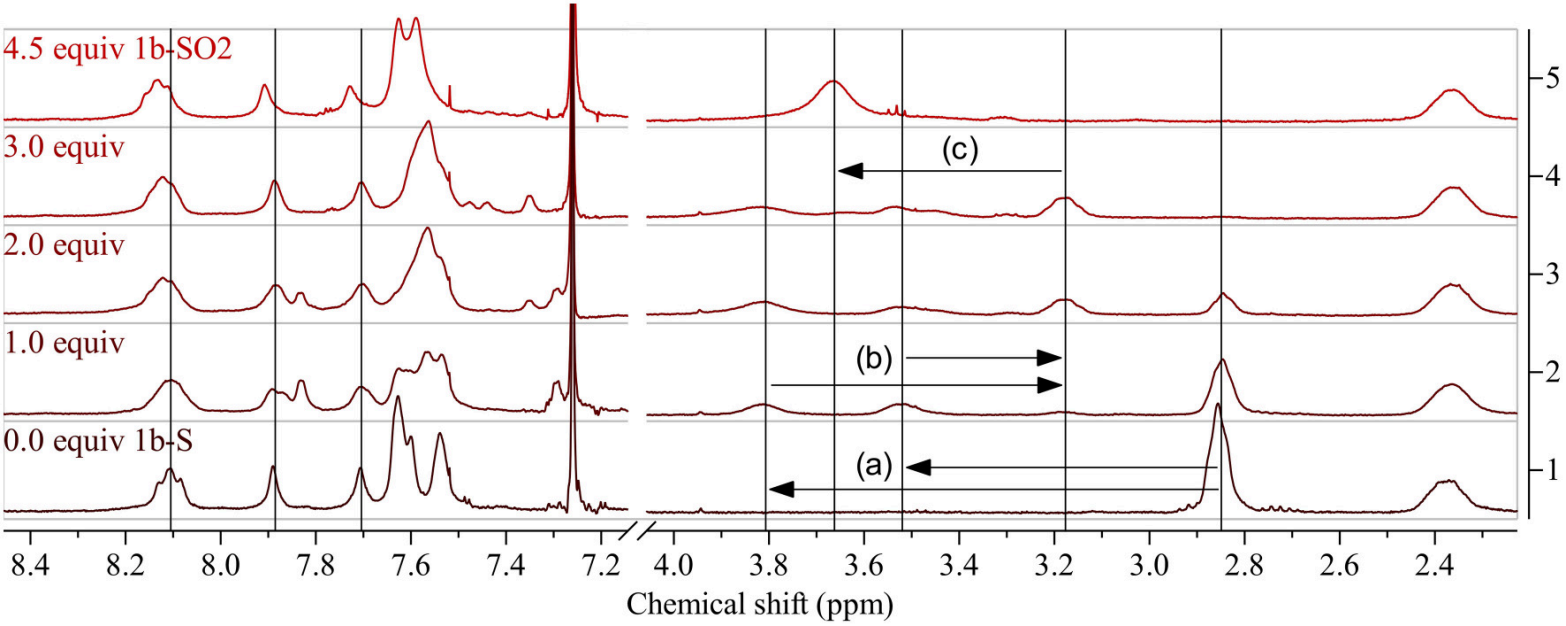
^1^H NMR spectra of **1b-S** (bottom) and corresponding polymers obtained by modification using increasing amounts of DMDO (bottom to top; 4.5 equiv: **1b-SO**_**2**_). Arrows show the shifts of the peak of the aliphatic protons next to the thioalkyl sulfur upon modification: (a) downfield shift and splitting of the peak of the aliphatic protons next to the thioalkyl sulfur upon oxidation to the sulfoxide, (b) upfield shift and reunification by further oxidation to the sulfone, (c) downfield shift upon occurrence of two neighboring sulfones. See Supplementary Material for full spectra.

Post-polymerization modification of **1b-S** was carried out under the same conditions (Table 2 gives an overview of the experiments). However, as there are twice as many thioalkyl groups per repeat unit present, 4.5 equiv DMDO were used for the oxidation to **1b-SO**_**2**_ (Figure 2, top right). As for the modifications of **1a-S**, the peak of the aliphatic protons next to the sulfur atom shifted significantly, from 2.85 to 3.66 ppm (Figure 4). As there are no protons present at the benzothiadiazole units, shifts in the aromatic region were less pronounced.

**Table 2:** Screening of reaction conditions using PCDTBT-based polymers 1a-S and 1b-S as starting polymers.

**Starting polymer**	**Reaction solvent**	**DMDO [equiv]**	**Reaction time**	**Result (analysis by ^**1**^H NMR spectroscopy, also see Figures 3, 4)**
**1a-S**	CH_2_Cl_2_	2.5	1.5 h	Precipitation of the polymer upon oxidation, byproduct peaks at 9.17 and 8.55 ppm
**1a-S**	Chlorobenzene	0.5	1.0 h	Partial oxidation to SO, no formation of SO_2_
**1a-S**	Chlorobenzene	1.0	1.0 h	Complete and selective oxidation to **1a-SO**, no formation of SO_2_
**1a-S**	Chlorobenzene	1.5	1.0 h	SO_2_:SO ratio: approx.1:1, no byproduct signal
**1a-S**	Chlorobenzene	2.0	1.0 h	Incomplete oxidation to SO_2_ (SO_2_:SO ratio: 9:1)
**1a-S**	Chlorobenzene	2.5	1.0 h	Complete oxidation to **1a-SO**_**2**_, very small byproduct peak at 9.17 ppm, high selectivity
**1a-S**	Chlorobenzene	3.0	24 h	Complete oxidation to SO_2_, but byproduct peaks at 9.17 and 8.55 ppm
**1b-S**	Chlorobenzene	1.0	1.0 h	Formation of SO, also some SO_2_
**1b-S**	Chlorobenzene	2.0	1.0 h	Considerable formation of SO_2_, S still present
**1b-S**	Chlorobenzene	3.0	1.0 h	Formation of some neighboring SO_2_, no more S
**1b-S**	Chlorobenzene	4.5	1.0 h	Complete oxidation to **1b-SO**_**2**_

*Final conditions for the synthesis of 1a-SO, 1a-SO_2_, and 1b-SO_2_ highlighted in gray. Reaction procedure: DMDO in acetone (0.074 M) added to stirred polymer solutions (concentration of repeat unit: 0.01 M) at room temperature. Work-up: evaporation of solvents (and excessive DMDO) under reduced pressure at 50°C*.

Using 1.0 equiv DMDO for the modification, the aliphatic peak split into two peaks at 3.81 and 3.52 ppm (Figure 4(a)), again confirming the formation of sulfoxides. The weak peak at 3.18 ppm is assigned to some oxidation to sulfones, indicating that the modification to sulfoxides is less selective for **1b-S** than for **1a-S**. Using 2.0 equiv, this peak increased to the same size as the residual peak of the non-oxidized thioalkyl substituents, which is in accordance with the assignment of the peaks. The peak of the thioalkyl substituents disappeared when using 3.0 equiv; the peak of the final polymer **1b-SO**_**2**_ at 3.66 ppm (attributed to two neighboring sulfone groups) started to appear.

### F8BT-Based Polymers

**Figure 5:**
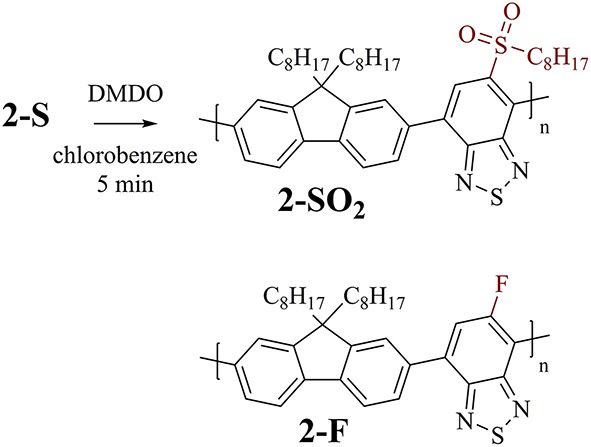
Top: Post-polymerization modification of F8BT-based polymer **2-S** to sulfone-substituted polymer **2-SO**_**2**_. Bottom: Related F8BT-based polymer **2-F** (without thioalkyl substituents) used for control experiments.

F8BT-based polymer **2-S** (Figure 5, top) was used for investigating the time required for complete oxidation to **2-SO**_**2**_ under the same conditions as used for the modifications of the PCDTBT-based polymers. An overview of the experiments is provided in Table 3.

**Table 3:** Screening of reaction times using F8BT-based polymer **2-S** as starting polymer.

**Starting polymer**	**DMDO [equiv]**	**Reaction time**	**Result (analysis by ^**1**^H NMR spectroscopy, also see Figure 6)**
**2-S**	2.5	0 min	Incomplete oxidation
**2-S**	2.5	5 min	Complete and selective oxidation to **2-SO**_**2**_
**2-S**	2.5	1 h	Complete and selective oxidation to **2-SO**_**2**_
**2-S**	3.0	24 h	Complete and selective oxidation to **2-SO**_**2**_ (no byproduct signals)
**2-F**	2.5	5 min	No reaction, no change of ^1^H NMR spectrum

*Final conditions for the synthesis of **2-SO**_**2**_ highlighted in gray. The selectivity was investigated by treating **2-S** with a large excess of DMDO for prolonged time (3.0 equiv, 24 h) and by control experiments using **2-F**. Reaction procedure: DMDO in acetone (0.104 M) added to stirred polymer solutions in chlorobenzene (concentration of repeat unit: 0.01 M) at room temperature. Work-up: evaporation of solvents (and excessive DMDO) under reduced pressure at 50°C*.

**Figure 6:**
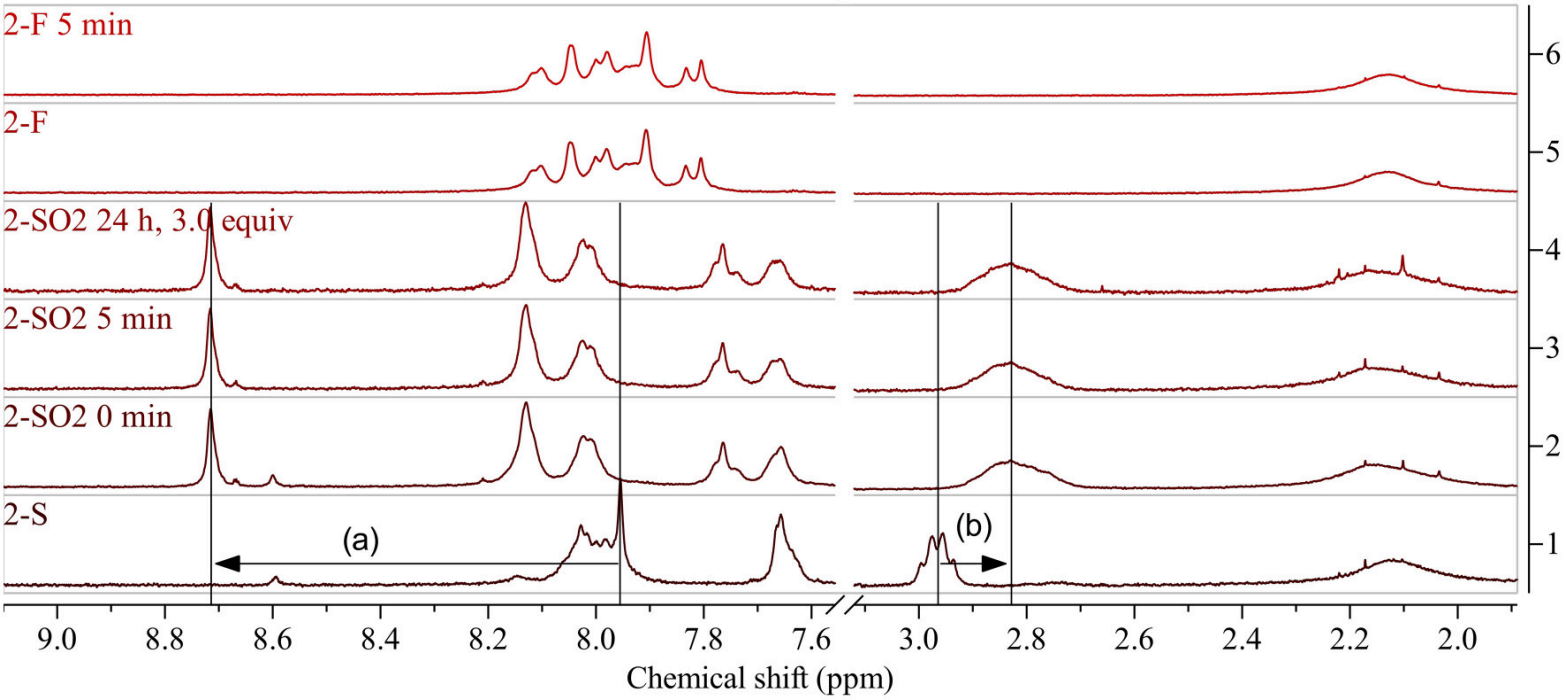
^1^H NMR spectra of **2-S** (bottom) and **2-SO**_**2**_ obtained by modification with DMDO for 0 min and 5 min before work-up (above). Arrows show indicative shifts upon modification. The selectivity of the modification was evaluated by treating the starting polymer with a large excess of DMDO for prolonged time (24 h, 3.0 equiv). Further control experiments were carried out by treating **2-F** with DMDO for 5 min, resulting in no change of the ^1^H NMR spectrum (top). Arrows show indicative shifts upon modification: (a) downfield shift of the peak of the benzothiadiazole proton by oxidation to the sulfone and (b) upfield shift of the aliphatic protons next to the thioalkyl sulfur upon oxidation to the sulfone. See Supplementary Material for full spectra.

A reaction time of 1 h (before evaporation of solvents and excessive reagent) resulted in complete oxidation to **2-SO**_**2**_, indicated by shifts of the protons next to the sulfur atom from 7.95 to 8.71 ppm (benzothiadiazole proton, Figure 6(a)) and 2.96 to 2.83 ppm (aliphatic protons, Figure 6(b)). Interestingly, when reducing the reaction time to 5 min, the same results were obtained. Addition of DMDO to the reaction and immediate evaporation of solvents and reagent (0 min reaction time) resulted in incomplete oxidation.

In contrast to PCDTBT-based polymer **1a-S**, no side reactions were observed when treating **2-S** with 3.0 equiv DMDO for 24 h. The absence of side reactions was further confirmed for this type of polymer by treating **2-F** (Figure 5, bottom) with DMDO, which did not result in any changes of the ^1^H NMR spectrum.

## Materials and Methods

Solvents and chemicals were purchased from commercial suppliers and used without further purification. DMDO was prepared scaling down a published protocol (Mikula et al., 2013).

### Synthesis of Monomer 3-S and PCDTBT-Based Polymers 1a-S, 1b-S, and 1a-SO_2_-Preox

Monomer **3-S** and polymer **1a-S** were synthesized according to our previously reported work (Creamer et al., 2017), as was **1b-S** (Casey et al., 2014).

Synthesis of **1a-SO**_**2**_**-preox** was based on these two previously reported works. Monomer **3-SO**_**2**_ (63.5 mg, 0.100 mmol, 1.0 equiv), 9-(9-heptadecanyl)-9*H*-carbazole-2,7-diboronic acid bis(pinacol) ester (65.8 mg, 0.100 mmol, 1.0 equiv), Pd(PPh_3_)_4_ (2.3 mg, 0.002 mmol, 0.02 equiv), and a stirrer bar were added to a 5 mL high-pressure vial. The vial was sealed with a septum and flushed with argon, before degassed toluene (1.4 mL), degassed aqueous 1 M Na_2_CO_3_ (0.32 mL, 0.32 mmol, 3.2 equiv), and 1 drop of Aliquat 336 were added. The whole solution was then degassed again for 40 min before the reaction was heated to 120°C for 1 day. The reaction was cooled to room temperature, precipitated in methanol, stirred for 30 min and filtered through a Soxhlet thimble. The polymer was then extracted (Soxhlet) using methanol, acetone, hexane, and chloroform in that order. The chloroform fraction was collected and evaporated to yield polymer **1a-SO**_**2**_**-preox** (86 mg, 97%). ^1^H NMR in accordance with **1a-SO**_**2**_; M_n_: 53 kDa, M_w_: 97 kDa, Ð: 1.8.

### Synthesis of F8BT-Based Polymers 2-F and 2-S

**2-F** was synthesized according to our previously reported work (Creamer et al., 2018).

Synthesis of **2-S** was based on our previously reported method (Creamer et al., 2018). **2-F** (94 mg, 0.174 mmol, 1.0 equiv), octanethiol (0.1 mL, 0.58 mmol, 3.3 equiv) and K_2_CO_3_ (240 mg, 1.74 mmol, 10.0 equiv) were added to a 5 mL high-pressure vial. The vial was sealed with a septum and degassed with argon, before anhydrous chlorobenzene (3 mL) and DMF (1 mL) were added. The solution was heated at 130°C for 4 h. After cooling, the solution was precipitated into methanol. The suspension was stirred for 30 min, filtered and the precipitate was washed several times with methanol and acetone to remove unreacted thiol and residual DMF. The yellow solid was isolated and dried under vacuum (85 mg, 0.127 mmol, 73%). ^1^H NMR (400 MHz, CDCl_3_): 8.23–7.81 (m, 5 H), 7.66 (br, 2H), 3.05–2.87 (m, 2H), 2.43–1.84 (m, 4 H), 1.72–0.92 (m, 36H), 0.91–0.74 (m, 9 H) ppm.

### Synthesis of Monomer 3-SO_2_

To a stirred solution of starting material **3-S** (90 mg, 0.15 mmol, 1.0 equiv) in CH_2_Cl_2_ (1.5 mL, 0.1 M) was added DMDO (5.1 mL, 0.074 M in acetone, 0.38 mmol, 2.5 equiv) at r.t. The reaction stirred for 90 min and was then evaporated *in vacuo*. The residue was purified by flash chromatography (petroleum ether:CH_2_Cl_2_ 1:1) to afford pure monomer **3-SO**_**2**_ (75 mg, 0.12 mmol, 79%). ^1^H NMR (600 MHz, CDCl_3_): δ = 8.61 (s, 1H), 7.90 (d, *J* = 4.0 Hz, 1H), 7.38 (d, *J* = 3.9 Hz, 1H), 7.22 (d, *J* = 3.9 Hz, 1H), 7.21 (d, *J* = 4.0 Hz, 1H), 2.97–2.86 (m, 2H), 1.63–1.55 (m, 2H), 1.29–1.11 (m, 10H), 0.84 (t, *J* = 7.1 Hz, 3H) ppm. ^13^C(APT) NMR (150 MHz, CDCl_3_): δ = 155.85 (C), 152.52 (C), 139.91 (C), 139.16 (C), 133.67 (C), 133.48 (CH), 131.13 (CH), 130.44 (CH), 129.02 (CH), 127.25 (C), 125.32 (C), 123.07 (CH), 117.52 (C), 117.04 (C), 54.35 (CH_2_), 31.77 (CH_2_), 28.99 (CH_2_), 28.90 (CH_2_), 28.30 (CH_2_), 22.83 (CH_2_), 22.69 (CH_2_), 14.20 (CH_3_) ppm. HRMS (APCI/Orbitrap) m/z: [M]^+^ Calcd for C_22_H_22_Br_2_N_2_O_2_S_4_ 631.89254; Found 631.89270. See Supplementary Material for NMR spectra and HRMS results.

### General Procedure for Post-polymerization Modification

To a stirred solution in chlorobenzene (concentration of polymer repeat unit: 0.01 M) at r.t. was added DMDO (0.074 M or 0.104 M in acetone, amount per repeat unit given in Tables 2, 3). The reaction stirred at r.t. for the reaction time given in Tables 2, 3 and was then evaporated *in vacuo* to afford the modified polymer.

## Discussion

Both, PCDTBT- and F8BT-based thioalkyl-substituted polymers, could be modified very selectively using DMDO. Even when treating the polymers for 24 h with a large excess of DMDO, no side reactions were observed for F8BT-based polymer **2-S** and only very weak side reactions were found for PCDTBT-based polymer **1a-S**, which features carbazole and thiophene units potentially prone to oxidation. Oxidation to sulfoxides (instead of sulfones) was surprisingly selective for the investigated polymer **1a-S**, but not selective for **1b-S**, where two adjacent thioalkyl substituents are present.

Chlorobenzene was found to be a better solvent for the modifications than CH_2_Cl_2_, as the polymers stayed in solution when adding the reagent. Experiments in chlorobenzene revealed that the modification is a very fast reaction. Even when adding the reagent and immediately evaporating the solvent and reagent the reaction was almost complete. Complete modification was observed when stirring the reaction for 5 min before evaporation.

As our findings demonstrate, oxidation of thioalkyl substituents to sulfoxides and sulfones using DMDO is indeed a very useful reaction for the post-polymerization modification of conjugated polymers such as PCDTBT and F8BT. However, for applications requiring very high selectivity of the modification it should be considered that side reactions may take place at other sites prone to oxidation (e.g., carbazoles)—although these side reactions were found to be very slow in our experiments. Fluorene and benzothiadiazole units as in F8BT were found to be a safe choice to avoid all side reactions. For the preparation of sulfoxide-substituted polymers, it should further be considered that adjacent functional groups may impact the selectivity; oxidation to sulfones may occur before complete oxidation to sulfoxides.

We believe this post-polymerization modification method is a very useful tool for changing the properties of polymers drastically and for preparing electron-poor conjugated polymers. The modification may even allow for obtaining electron acceptors for solar cells directly from the electron donors. Furthermore, we envision exciting future applications of this modification ranging from surface modification of conjugated polymer nanoparticles to modification of polymer thin-films.

## Author Contributions

ER synthesized monomer **3-SO**_**2**_ and polymer **1a-SO**_**2**_**-preox** and carried out the modifications of PCDTBT-based polymers. AdC prepared **1a-S**, **2-S**, and **3-S** and contributed to writing the manuscript. SC and AbC prepared **2-F** and **1b-S**. SE carried out the modifications of F8BT-based polymers. MH designed and supervised experiments and contributed to writing the manuscript. FG designed and supervised experiments, measured NMR spectra, and prepared the manuscript.

### Conflict of Interest Statement

The authors declare that the research was conducted in the absence of any commercial or financial relationships that could be construed as a potential conflict of interest.
