# Supplementary material for: Fast and Selective Post-polymerization Modification of Conjugated Polymers Using Dimethyldioxirane
Source: Front Chem. 2019 Mar 11;7:123. doi: 10.3389/fchem.2019.00123 (PMC6421264; doi:10.3389/fchem.2019.00123)
Supplement: Supplementary file 1 [file Data_Sheet_1.PDF]

Supplementary Material

Fast and selective post-polymerization modification of conjugated polymers using dimethyldioxirane

Emmanuel Reichsöllner, Adam Creamer, Shengyu Cong, Abby Casey, Simon Eder, Martin Heeney, Florian Glöcklhofer*

*** Correspondence:** Florian Glöcklhofer: f.glocklhofer@imperial.ac.uk

1 Instrumentation

NMR spectra were recorded on a Bruker AV-400 spectrometer (400 MHz) and a Bruker Avance DRX-400 spectrometer (400 MHz).

Mass average (M_w) and number average (M_n) molecular weights were determined with an Agilent-Technologies 1260 Infinity GPC System with 1260 RID and DAD VL attachments. Measurements were performed at 80°C, using analytical grade chlorobenzene as eluent with two PLgel 10 µm MIXED B columns in series. Molar mass, as a function of elution time through the columns, was calibrated using known molecular weight Agilent EasiVial narrow dispersity polystyrene standards. Samples were prepared using analytical grade chlorobenzene in concentrations of ~1–2 mg mL^{-1} and filtered with VWR PES membrane 0.45 µm syringe filters before submission. An injection volume of 50 µL and GPC flow rate of 1.00 mL min^{-1} was used.

2 NMR spectra

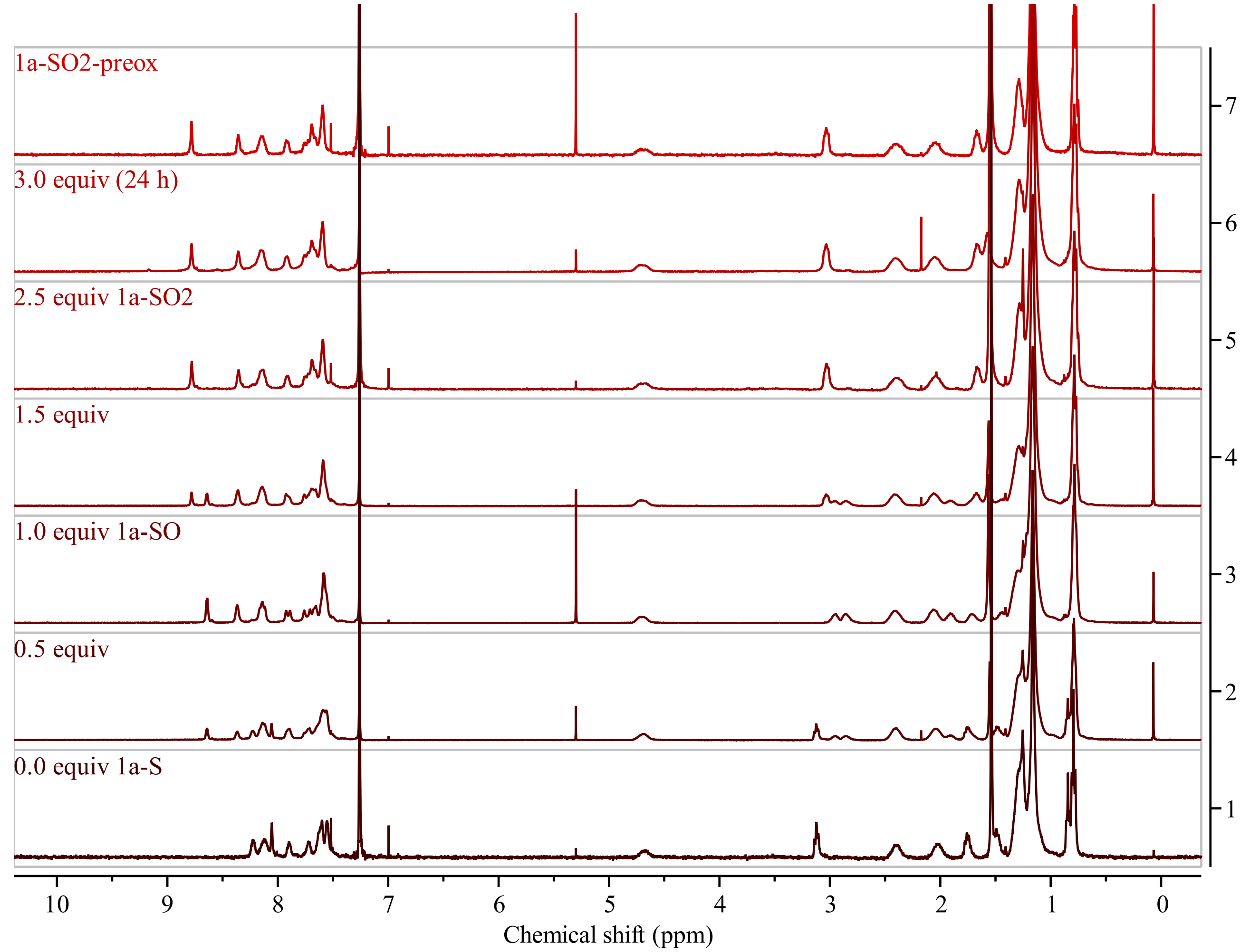

Supplementary Figure 1. ^{1}H NMR spectra of **1a-S**, the corresponding polymers modified with different amounts of DMDO, and **1a-SO$_2$-preox**.

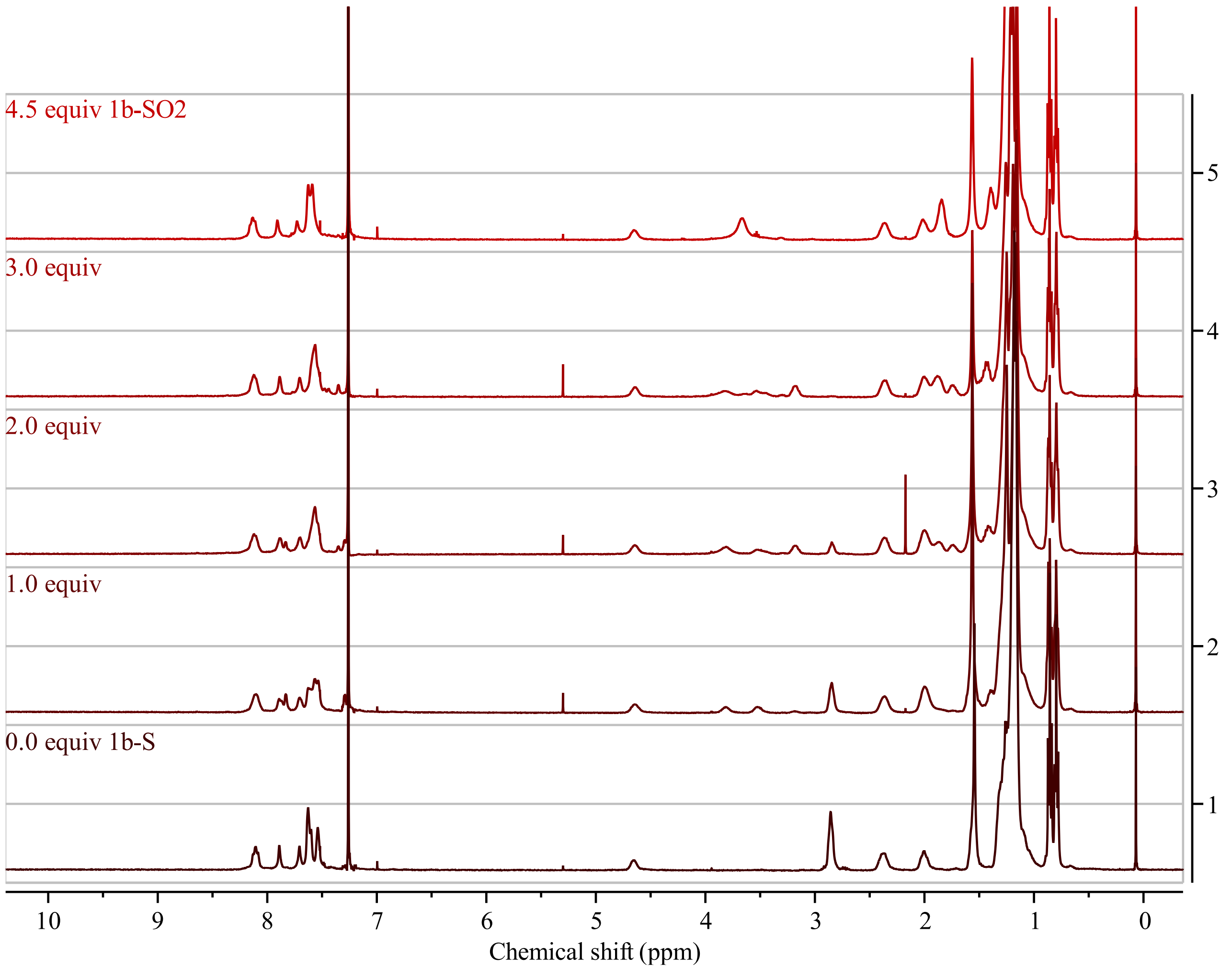

Supplementary Figure 2. ^{1}H NMR spectra of **1b-S** and the corresponding polymers modified with different amounts of DMDO.

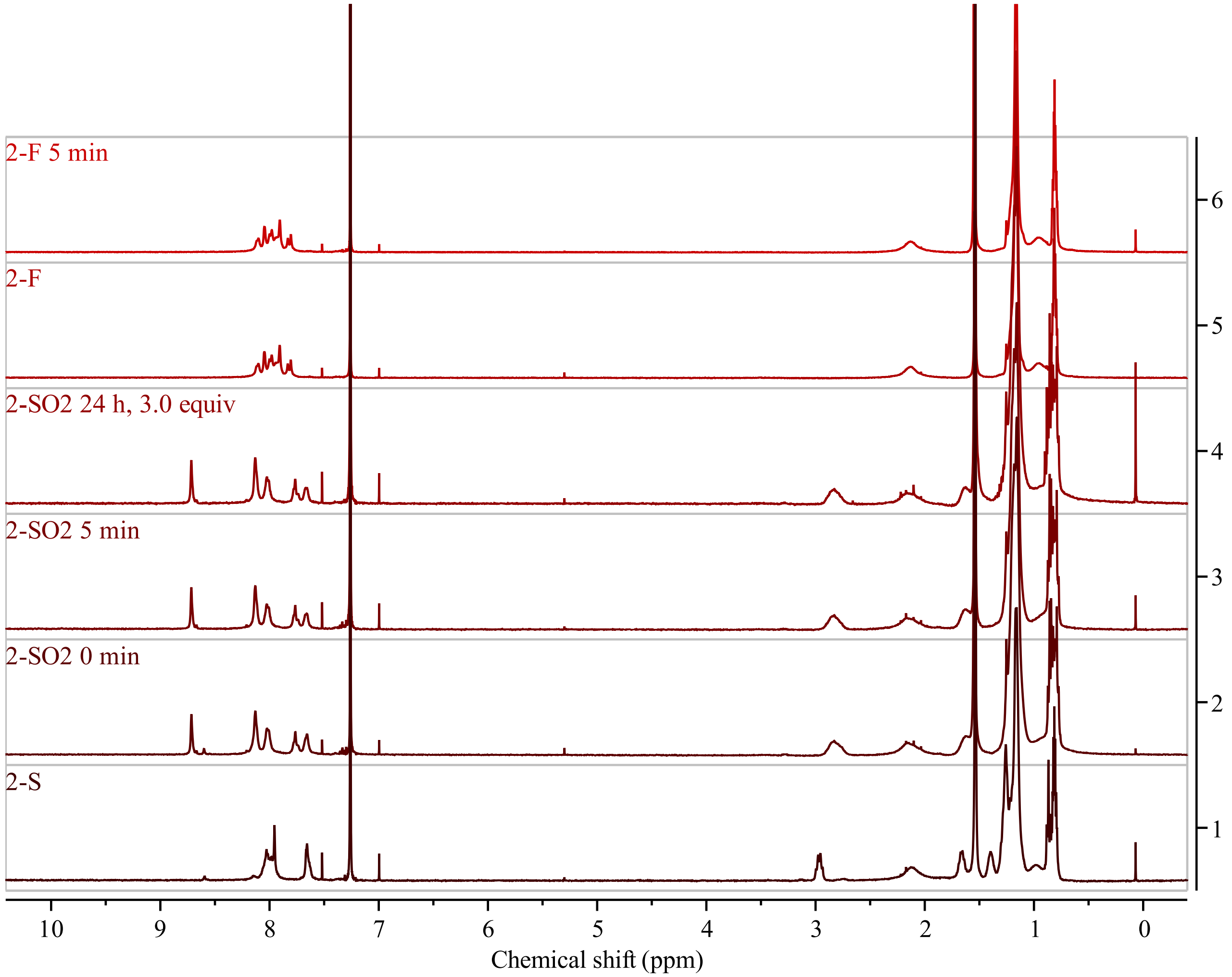

Supplementary Figure 3. ^{1}H NMR spectra of **2-S**, the corresponding polymers modified with DMDO for different times, **2-F**, and **2-F** treated with DMDO.

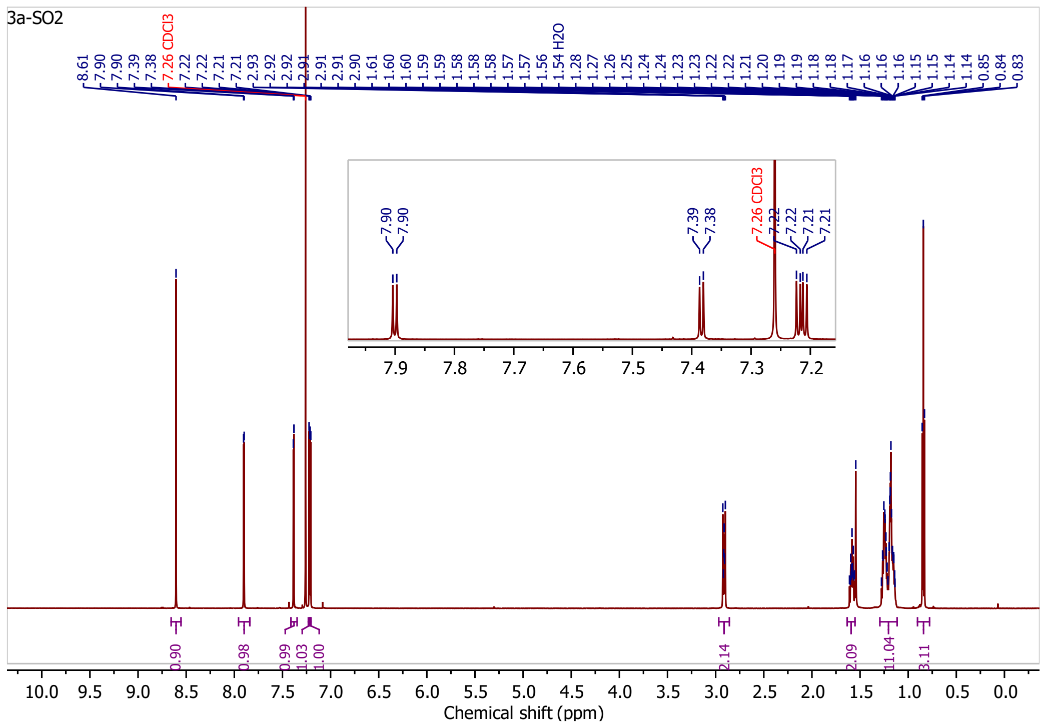

Supplementary Figure 4. ^{1}H NMR spectrum of monomer **3-SO$_2$**.

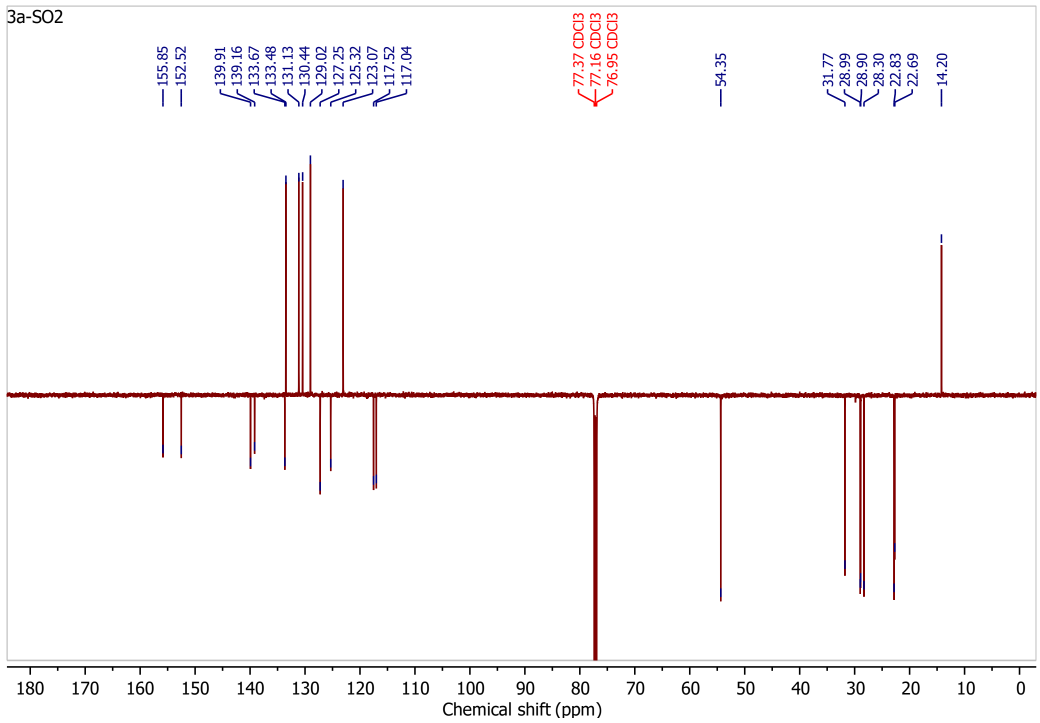

Supplementary Figure 5. ^{13}C(APT) NMR spectrum of monomer **3-SO$_2$**.

3 High-resolution mass spectrometry (HRMS)

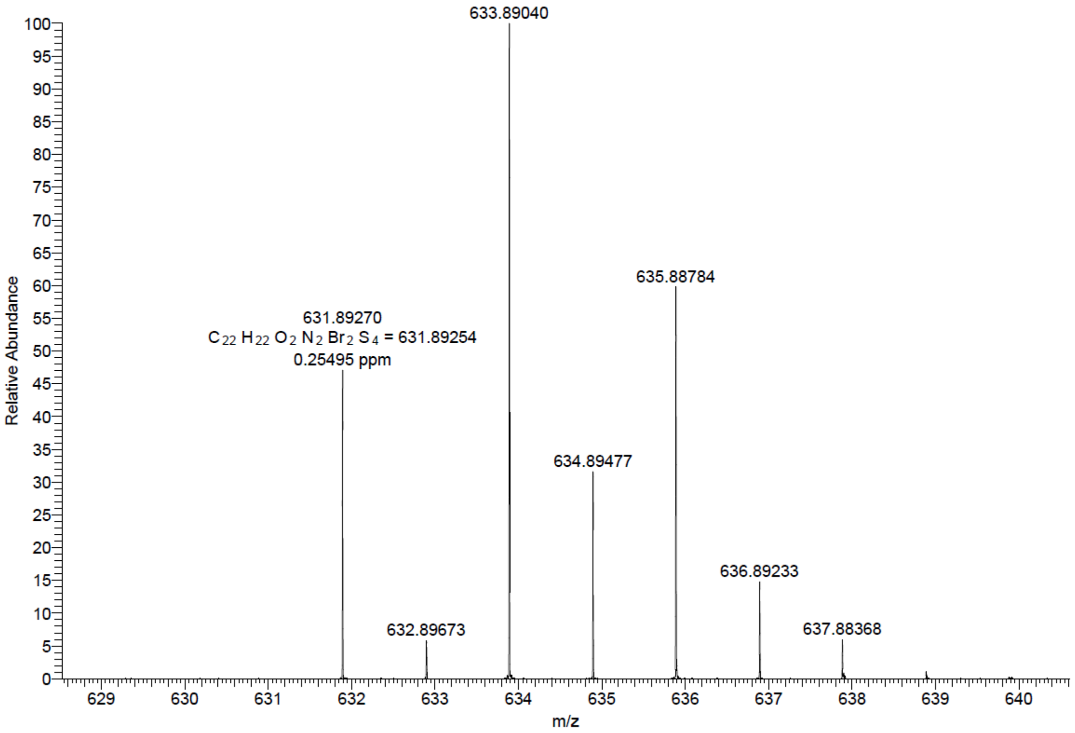

Supplementary Figure 6. HRMS measurement of monomer **3-SO_2**.
